# Pantoea dispersa Causing Bacteremia and Endocarditis in an Immunocompromised Adult: A Rare Case

**DOI:** 10.7759/cureus.74433

**Published:** 2024-11-25

**Authors:** Emma D Famous, Hannah Thompson, Amgad Masoud

**Affiliations:** 1 School of Medicine, University of Missouri Kansas City School of Medicine, Kansas City, USA; 2 Department of Internal Medicine, University of Missouri Kansas City School of Medicine, Kansas City, USA

**Keywords:** gram-negative bacteremia, immunocompromised patient, indolent, infective endocarditis, nidus, north america, novel presentation, pantoea dispersa

## Abstract

Gram-negative rods, namely, *Escherichia coli* and* Salmonella spp.*, are the most common causative agents of bacteremia. The genus *Pantoea*, another group of Gram-negative rods, is a relatively uncommon cause of bacteremia. Our literature review revealed only eight other cases of *Pantoea dispersa *infection in humans. In an otherwise healthy patient, *Pantoea* bacteremia is often transient and can be cleared with host immune defenses. Clearing of the bacteria may be complicated by an immunosuppressed state and infection with nidus. This is a case of bacteremia and endocarditis caused by *Pantoea dispersa* in an immunocompromised 75-year-old female.

## Introduction

*Pantoea dispersa* is part of the genus *Pantoea*, a group of Gram-negative, non-encapsulated, non-spore-forming straight rods that are ubiquitous in soil and cause plant infections. Most existing literature about this bacteria has been regarding plant-based research. Only eight previous cases of *Pantoea dispersa* infections have been reported worldwide. Of those cases, six were in adult patients. Three of the patients recovered, including bacteremia in one patient in Brazil, a case of peritoneal dialysis catheter infection causing bacteremia in a patient in Malaysia, and acute cholangitis with bacteremia in a patient in Japan [1-3]. Three patients did not survive; a patient with co-infection of disseminated fungal disease, a patient with respiratory infection, a patient with nosocomial-caused bacteremia, and one of the neonates with sepsis [4-6]. There are also two reported cases of* Pantoea dispersa* causing sepsis in neonates in Brazil [7]. One neonate recovered and the other did not survive [7]. An immunocompromised state is a common feature of known *Pantoea* infections [5,6]. In our literature review, we found no cases of *Pantoea dispersa* infection in a human in North America, and no cases causing endocarditis. Out of eight reported cases, only one case was caused by a nidus of an indwelling port.

## Case presentation

A 75-year-old female with a history of immune thrombocytopenia (ITP) and colon cancer, in remission since 2022, presented to outpatient Rheumatology followed by the Emergency Department, for cyclical fever and chills. Her ITP was being treated with mycophenolate mofetil (MMF) 1000 mg three times daily, hydroxychloroquine 200 mg daily, and prednisone 5 mg daily, all of which she takes chronically. She had undergone extensive treatment for colon cancer, including chemotherapy through a port placed in 2017 and a colonic resection. A colonoscopy completed in 2022 confirmed the colon cancer was in remission. The patient traveled to Mexico at the end of 2022 following her remission results. The chemotherapy port was still in place with plans to have it removed in 2023. She was hospitalized while in Mexico in the spring of 2023 with a reported platelet count of 44,000 and admitted for an ITP flare and was treated with a prednisone taper from 60 mg in addition to her home MMF and hydroxychloroquine. During her hospitalization, she reported having muscle twitching and body shakes along with chills and fever every time the chemotherapy port was accessed. The port was accessed for routine labs. The patient reported that blood cultures were drawn during her hospitalization and were negative. We were unable to attain her medical records from Mexico.

After her return to the United States in the fall of 2023, she presented to her rheumatologist’s office for follow-up. She reported to her rheumatologist that she had been having episodes of chills and body shakes for over six months. She also reported increased shortness of breath. Peripheral blood cultures taken from two sites were collected. The next day, preliminary results revealed a Gram-negative bacteremia, and she promptly presented to the emergency department for further management.

Upon presentation to the hospital, she endorsed a mild headache and some fatigue but otherwise reported feeling well. She was vitally normal and afebrile, and she was well-appearing without signs of distress. The physical exam was unremarkable. Initial lab tests, including basic metabolic panel (BMP), complete blood count (CBC), liver function test (LFT), C-reactive protein (CRP), erythrocyte sedimentation rate (ESR), and thyroid-stimulating hormone (TSH) were unremarkable. A chest X-ray and abdominopelvic CT with contrast did not show any signs of infection. A second set of blood cultures was drawn, one from the right wrist and the other through her chemotherapy port. Given her immunocompromised status, despite her well-appearing presentation, she was treated with empiric cefepime after the repeat cultures were drawn. General surgery removed the chemotherapy port on hospital day two.

The first set of blood cultures, taken before the start of IV antibiotics, grew *Pantoea dispersa*. Of the second set of blood cultures drawn on hospital day two, the one drawn through the port grew *Pantoea dispersa* while the culture drawn from the right wrist had no bacterial growth at five days. The port was sent for culture after removal on hospital day 2. An aerobic culture showed no growth after five days. However, an anaerobic culture was unable to be performed. By hospital day three, three days after the start of antibiotics, cultures drawn from bilateral upper extremities were both negative. Susceptibility testing showed that *Pantoea dispersa* was sensitive to all antibiotics tested except cefazolin. See Table 1 for more detailed susceptibility results. The antibiotic treatment was changed to ceftriaxone, as it allowed for once daily intravenous dosing. A trans-thoracic echocardiogram (TTE) was inconclusive so a trans-esophageal echocardiogram (TEE) was performed which showed suspicious vegetation on the mitral valve. The patient did well throughout her hospital course and remained largely asymptomatic. Following antibiotic initiation, she had an increased absolute neutrophil count to 18.2 that resolved over her seven-day hospital stay. Given her history of ITP, her platelets were closely monitored throughout her stay and remained within normal limits. All immunosuppressive treatment was withheld except for prednisone 5 mg daily during her hospitalization. A peripherally inserted central catheter (PICC) line was placed for her to receive six weeks of ceftriaxone at home.

**Table 1:** Pantoea dispersa susceptibilities

Blood Culture Bacterial		
	MIC Dilution	MIC Interpretation
Cefazolin	16	I
Cefepime	<=1	S
Ceftriaxone	<=1	S
Gentamicin	<=1	S
Levofloxacin	<=0.12	S
Meropenem	<=0.25	S
Pipercillin/Tazobactam	<=4	S
Tobramycin	<=1	S
Trimethoprim/Sulfamethoxazole	<=20	S
MIC = Minimum Inhibitory Concentration; I = Intermediate Susceptibility; S = Susceptible		

The patient presented to her outpatient follow-up appointments, including Internal Medicine, Rheumatology, and Infectious Disease appointments, at one, three, and six weeks after discharge, respectively. At these visits, she reported the resolution of her presenting symptoms, indicating improvement and resolution of her bacteremia and endocarditis. She completed her course of antibiotics and the PICC line was removed. Rheumatology re-initiated and managed her immunosuppressive medications. The patient originally declined to complete a follow-up transthoracic echocardiogram (TTE) or transesophageal echocardiogram (TEE). She later completed a TTE in the spring of 2024, which showed no evidence of endocarditis.

## Discussion

This is a case of a 75-year-old female with indolent *Pantoea dispersa* bacteremia and endocarditis caused by an infected chemotherapy port. Her course was complicated by her immunocompromised status and the long-standing, indolent nature of the infection, which allowed the patient to live mostly symptom-free with only a few short episodes of intermittent fever and chills for many months. The port was utilized for routine labs. Every time, her symptoms of fever and chills were correlated to port access. This case is unique in its epidemiology, causative agent, route of infection, and presentation of endocarditis, all of which we will discuss here. First, causative epidemiology and causative agent. We were able to find only eight other cases of *Pantoea dispersa* in our literature review, highlighting the rarity of *Pantoea dispersa* as an infectious agent. These cases encompass hematologic, abdominal, and respiratory infections throughout Asia, Brazil, and Germany [1-7]. None of these cases were published in North America [1-7]. This patient resides in the United States and had an extended stay in Mexico. Second, the route of infection. The nidus of infection was the chemotherapy port. The route by which this port was infected is unknown but potential causes include the long-standing nature of the chemotherapy port, non-sterile access of the port, and exposure of the port to soil contamination where the bacteria is ubiquitous. One out of the eight reported cases was associated with an indwelling port [5]. Our case differs, as this was a central line catheter, rather than a peritoneal dialysis catheter [5]. Lastly, none of the previously published reports of* Pantoea dispersa* have reported endocarditis [1-7]. Our case is also unique in the timing of her symptoms. She was medically stable and without a flare of her ITP for more than six months. She was only symptomatic with intermittent fever and chills upon port access. Our patient responded well to antibiotic therapy, similar to half of the other reported *Pantoea dispersa* cases [1-3,7]. It is widely known and accepted that the most common cause of endocarditis is Gram-positive bacteria, specifically *Staphylococcus, Streptococci, *and* Enterococci*. While Gram-negative rods are common causes of bacteremia, they are less often causes of endocarditis [8,9]. *Pantoea dispersa* is a Gram-negative bacteria, further distinguishing the uniqueness of this case. Imaging in this case was suspicious for endocarditis, and while the patient was treated with six weeks of antibiotics, she originally declined to complete a follow-up TTE. She later underwent TEE for unrelated reasons, which was without signs of endocarditis, indicating resolution with antibiotic treatment.

## Conclusions

We present a case of *Pantoea dispersa* bacteremia and endocarditis caused by an indwelling port acting as a nidus. The patient, a 75-year-old female of immunocompromised status, presented with symptoms of cyclical fever and chills, which correlated to port access, which was utilized for routine labs. A literature search was performed, and there have only been eight reported cases of *Pantoea dispersa* bacteremia in humans worldwide. There are no other cases of *Pantoea dispersa *infection in a human in North America, as well as no other known reported case causing endocarditis. 

In addition, this case highlights the importance of proper port maintenance, awareness of complications, and evaluating the necessity of their prolonged use, especially in immunocompromised populations. While rare, *Pantoea dispersa* infections continue to be reported, and it has been proven that it can infect a variety of organ systems. It should be kept in mind as a causative agent, especially considering this new epidemiology with our case in North America.
